# Hagfish to Illuminate the Developmental and Evolutionary Origins of the Vertebrate Retina

**DOI:** 10.3389/fcell.2022.822358

**Published:** 2022-01-26

**Authors:** Sarah N. Bradshaw, W. Ted Allison

**Affiliations:** Department of Biological Sciences, University of Alberta, Edmonton, AB, Canada

**Keywords:** visual system, adult neurogenesis, Agnatha, cyclostome, eye development, vertebrate evolution, chordate, ciliary marginal zone

## Abstract

The vertebrate eye is a vital sensory organ that has long fascinated scientists, but the details of how this organ evolved are still unclear. The vertebrate eye is distinct from the simple photoreceptive organs of other non-vertebrate chordates and there are no clear transitional forms of the eye in the fossil record. To investigate the evolution of the eye we can examine the eyes of the most ancient extant vertebrates, the hagfish and lamprey. These jawless vertebrates are in an ideal phylogenetic position to study the origin of the vertebrate eye but data on eye/retina development in these organisms is limited. New genomic and gene expression data from hagfish and lamprey suggest they have many of the same genes for eye development and retinal neurogenesis as jawed vertebrates, but functional work to determine if these genes operate in retinogenesis similarly to other vertebrates is missing. In addition, hagfish express a marker of proliferative retinal cells (*Pax6*) near the margin of the retina, and adult retinal growth is apparent in some species. This finding of eye growth late into hagfish ontogeny is unexpected given the degenerate eye phenotype. Further studies dissecting retinal neurogenesis in jawless vertebrates would allow for comparison of the mechanisms of retinal development between cyclostome and gnathostome eyes and provide insight into the evolutionary origins of the vertebrate eye.

## 1 Introduction

To survive in adverse environments organisms must be able to perceive and respond to their surroundings. One of the most complex and remarkable sensory systems utilized in the animal kingdom is vision. Through specialized cells (photoreceptors) animals can make use of light information to sense their environment (Arendt, 2003). Across Metazoa, photoreception abilities range from simple light detection to the ability to form images. The eye allows for detection and processing of detailed light signals. Representatives of Cnidaria, Mollusca, Annelida, Onychophora, Arthropoda and Chordata are examples of organisms that have developed complex eyes, often associated with image formation (Zuker, 1994). Amazingly, despite these groups being phylogenetically distant, their eye structures appear to have independently evolved to perform similar functions (often through similar mechanisms) reinforcing the value of visual information across organisms (Fernald, 2000; Nilsson, 2013). With rare exception, vertebrates possess a complex camera-style eye that is remarkably conserved in form and function throughout the group. The retina of the vertebrate eye is specialized to form detailed images, detect motion, enhance contrast and/or perceive color (Yokoyama, 2000; Stenkamp, 2015; Morshedian and Fain, 2017). This allows vertebrates to find food and mates, avoid predation and navigate their surroundings across a variety of habitats. Despite the eye and retina being of primary importance to vertebrate ecology, a comprehensive appreciation of their evolutionary origins is lacking.

Contributing to the struggle to understand vertebrate eye evolution is the complexity of the organ itself. To receive light signals the retina contains highly specialized photoreceptor cells for light detection (these cells require a mechanism for light transduction and supporting cells to maintain their function) (Lamb et al., 2007; Fain et al., 2010). The light signals must then be transmitted to (and subsequently processed within) the brain for an animal to perceive the environment within their visual field. The communication and processing of this information requires an extremely complex neural network. Vertebrates employ multiple neuronal cell types to transmit and initiate processing of light signal information within the visual system (Stenkamp, 2015). In addition, the structure of the camera-style eye is also optimized to direct light onto the retina (to increase visual acuity). Structures such as the pupil and lens focus incoming light onto the retinal photoreceptors, pigment provides directionality for light signals and extraocular muscles allow vertebrates to shift their eye position. Many scientists, including and prior to Darwin, have pondered how these individual components may have arisen and come together through evolution to produce such a multiplex/sophisticated sensory organ (Fernald, 2004; Gehring and Seimiya, 2010).

**FIGURE 1:**
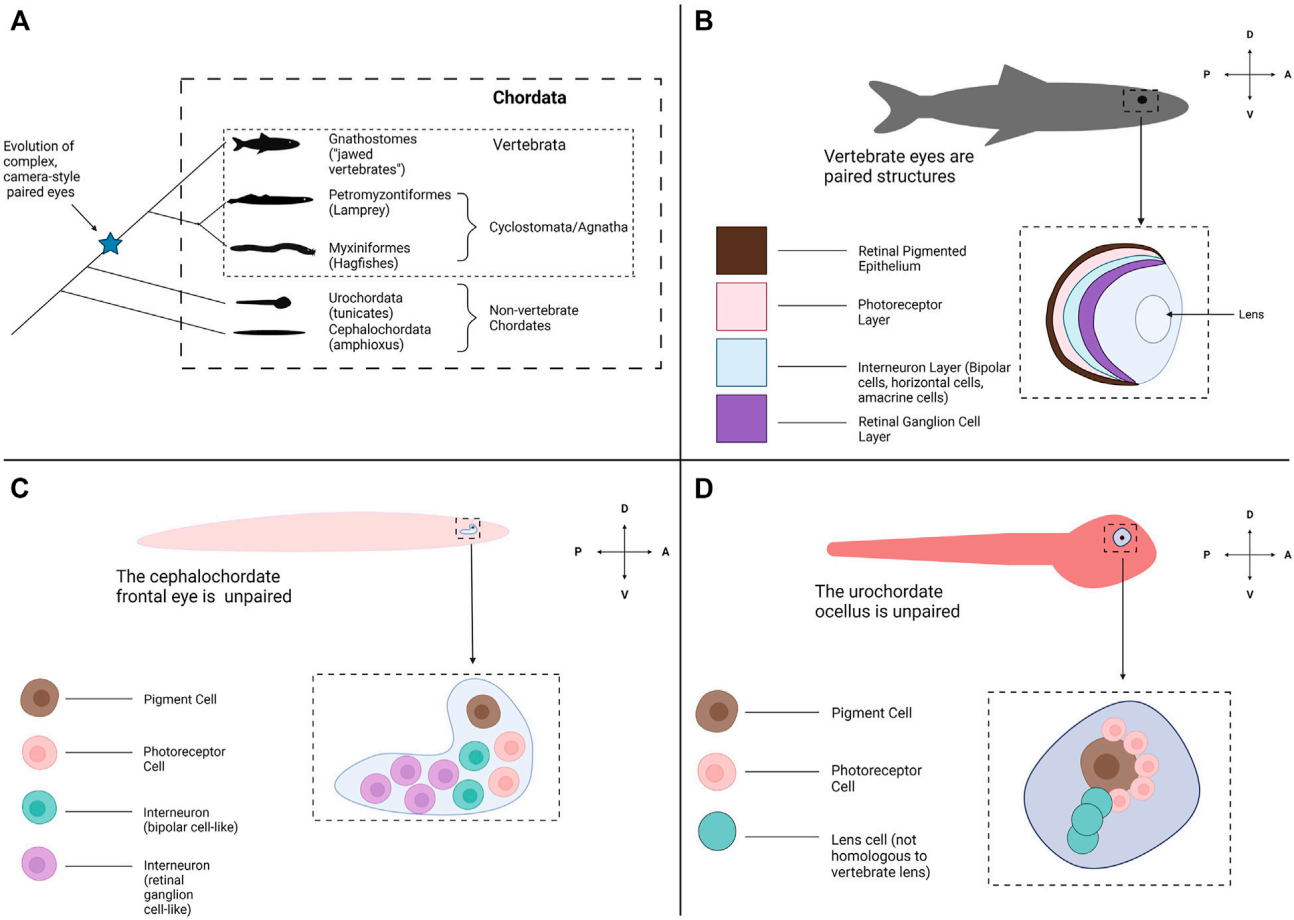
Evolutionary origins of the vertebrate eye and the appearance of other photoreceptive organs across Chordata. **(A)** A phylogeny of Chordata. Chordata consists of three subphyla: Cephalochordata (amphioxus), Urochordata (tunicates) and Vertebrata (the Vertebrates). Two clades form Vertebrata: the Hagfish and Lamprey (Myxiniformes and Petromyzontiformes) form a distinct group known as Cyclostomata (extant members of the jawless vertebrates (Agnatha)) and the jawed vertebrates form Gnathostomata. Paired, complex eyes appear within Vertebrata but not in the other Chordate subphyla. **(B)** Most all gnathostomes have the familiar vertebrate eye and retinal structures, conserved from fish through mammals. The eyes are paired and bilateral. Each eye contains a multi-layered retina for light detection and image formation, a pigmented retinal epithelial layer and a lens to focus incoming light. **(C)** The amphioxus frontal eye is an unpaired photoreceptive organ located at the anterior end of the amphioxus. The frontal eye contains a single pigment cell, several photoreceptor cells, and several interneurons (Vopalensky et al., 2012). **(D)** The tunicate ocellus is an unpaired photoreceptive organ that consists of a large pigment cell and associated photoreceptor cells (Horie et al., 2005). In some species the ocellus also contains lens cells, but these are not believed to be homologous with the lens structure of the vertebrate eye. Graphics created using BioRender.com.

Another factor confounding a complete understanding of eye evolution is how abruptly the eye appears within the vertebrate phylogeny and the lack of transitional states (Lamb et al., 2007) (Figure 1A). Almost all extant vertebrates possess a complete camera-style eye with a laminated/organized retina (Figure 1B). In contrast, the closest living relatives of the vertebrates, the cephalochordates (amphioxus) and urochordates (tunicates) do not have visual structures resembling a camera-style eye (Figures 1C,D). Instead, these groups possess comparatively simple clusters of photoreceptive cells associated with pigment cells. This leaves a gap around the chordate/vertebrate transition where eyes may have arisen (Figure 1A). Assuming the last common ancestor of vertebrates and other chordates had simple unpaired photoreceptive organs similar to extant non-vertebrate chordates, emergence of the vertebrate eye would have required numerous evolutionary innovations. This includes the development of structures to receive photic stimuli with increased resolution (i.e., a lens), directionality (i.e., the retinal pigmented epithelium) and image formation (i.e., complex photoreceptors) abilities (Nilsson, 2009). Without obvious intermediate stages, we can only hypothesize how these innovations may have taken place in the ancestral vertebrate. Some groups have examined fossil evidence from extinct species to try to find a transitional or early form of the vertebrate eye. However, many types of tissue do not fossilize well making fossil eye data difficult to interpret (Clements et al., 2016; Gabbott et al., 2016).

A promising avenue for exploring the origin of vertebrate eyes is to study an obscure but evolutionarily important group, the jawless vertebrates (Agnatha). The jawless vertebrates consist of two extant groups of anguilliform (elongate, eel-like in body form and swimming style) fishes, the lamprey and hagfish. These organisms are unique among extant vertebrates for lacking jaws and paired fins, and possess many distinct features which are considered “primitive”/ancestral compared to jawed vertebrates; whether these features truly represent the ancestral condition for vertebrates or are derived traits that arose in the cyclostome lineage is still a matter of debate (Kuratani and Ota, 2008; Shimeld and Donoghue, 2012). Lamprey are fishes that begin life as sediment dwelling filter feeders (Osório and Rétaux, 2008). Depending on the species, after metamorphosis lamprey can either become parasitic, feeding on the blood and tissue of aquatic organisms, or non-parasitic (generally do not feed as adults). Lamprey occur in both fresh and salt water environments. In contrast, hagfish are marine scavengers that live in the ocean depths and feed mostly on dead or dying animals (some sources suggest they may be more predatory than traditionally thought) (Martini, 1998). Various molecular and morphological studies agree that these groups comprise their own monophyletic clade, Cyclostomata forming a sister group to the jawed vertebrates (Gnathostomata) (Mallatt and Sullivan, 1998; Kuraku et al., 1999; Heimberg et al., 2010; Ota et al., 2011; Miyashita et al., 2019). The cyclostomes diverged from other vertebrates around 600 mya placing the group at a unique phylogenetic position that may be useful for unravelling the origins of the vertebrate eye and retina, especially when contrasted against the gnathostome condition (Figure 1A) (Blair and Hedges 2005). Despite the importance of these species to appreciating the evolutionary origins of the vertebrate eye, eye/retinal development in cyclostomes has been poorly studied.

The morphology of lamprey and hagfish eyes are important points of comparison for the eyes of other vertebrates. Adult lamprey have well-developed camera-style eyes that are similar to those found in jawed vertebrates (gnathostomes) (Lamb et al., 2007). There are some nuanced differences in retinal organization and retinal cell morphology but many elements of the visual system of lamprey and gnathostomes appear conserved (Fain, 2020). In contrast, hagfish eyes are strikingly rudimentary. Their small eyes are buried under a layer of skin or muscle depending on the genera (Fernholm and Holmberg, 1975). Hagfish eyes also lack a lens and pigment (features found in the eyes of all other living vertebrates). Recently it has been shown that in at least some hagfish the retina contains all the cellular layers seen in other vertebrates (Dong and Allison, 2021), but this had been obscured because the retina is poorly laminated (compared to the four clearly defined retinal layers of other vertebrates) (Lamb, 2013; Dong and Allison, 2021). In addition, the hagfish eye was shown to grow throughout ontogeny with a proliferative region at the margin of the retina, similar to the ciliary marginal zone of gnathostomes (discussed in Section 2.3) (Dong and Allison, 2021). These findings have raised questions about whether the hagfish and lamprey could represent two distinct phases of vertebrate eye evolution, with hagfish showing a more ancestral vertebrate eye form and the lamprey possessing a more derived form. However, others have suggested that hagfish may have once possessed more complex eyes that regressed due to living as scavengers in dim-light environments (Fernholm and Holmberg, 1975; Dong and Allison, 2021). Under this theory the hagfish eye could have become reduced to its current state (due to lack of use) after the hagfish began living in the deep ocean, similarly to the loss of eyes in other vertebrates living in dim-light environments (i.e., troglobionts such as the cavefish) (Sifuentes-Romero et al., 2020). Another related model suggests that the hagfish eye condition is rudimentary because it is neotenic/paedomorphic (retains larval features). This idea has been based on the observation that in lamprey adult animals have complex eyes whereas larval lamprey possess simpler eye structures (with the larval lamprey eye spot being compared to the hagfish eye) (Lamb et al., 2007; Suzuki and Grillner, 2018). Under the paedomorphosis model, hagfish would have undergone a heterochronic shift resulting in loss of the transition from a larval/juvenile to adult eye condition. Based on the available evidence it is difficult to rule out any of these possibilities.

To gain insight into the evolution of vertebrate eyes, we can take advantage of the available morphological, genetic and developmental information on eye formation in the cyclostomes. However, this type of information, especially that of retinal development, is lacking for both lamprey and hagfish. Both groups are non-model organisms and present unique challenges for study. Several studies have characterized the morphology and cell types within the lamprey eye (including the transition from larval to adult eye) but few studies have examined ocular gene expression during eye development (Meyer-Rochow and Stewart, 1996; Suzuki and Grillner, 2018; Fain, 2020). Further characterization of retinal and eye developmental pathways within lamprey could be used to strengthen (or refute) the comparisons made between lamprey and gnathostome eyes. Hagfish are even less studied. In addition to many species being deep-water, marine animals, historically hagfish embryos have been difficult to attain, making development difficult to study in this group (Gorbman, 1997). However, several groups have begun to successfully breed hagfish in a laboratory setting, opening future opportunities to study the embryonic hagfish eye (Holland, 2007; Ota and Kuratani, 2008). Within the current literature, studies have characterized the morphology of adult hagfish eyes and photoreceptors (Holmberg, 1970, Holmberg, 1971; Fernholm and Holmberg, 1975). More recently, hagfish retinal cell types have been studied through gene expression markers (Dong and Allison, 2021). Yet, information on how the hagfish eye develops is still highly lacking. To compare the cyclostome eye condition to the gnathostome condition, more data on cyclostome eye and retina development are necessary. Therefore, the goal of this review is to emphasize what is currently known about eye development in cyclostomes and to discuss how the comparison of retinogenesis in cyclostomes and gnathostomes would produce valuable information for exploring the origin of the vertebrate eye.

## 2 The Anatomy of Hagfish Eyes Is Rudimentary Compared to Lamprey and Gnathostomes

**FIGURE 2:**
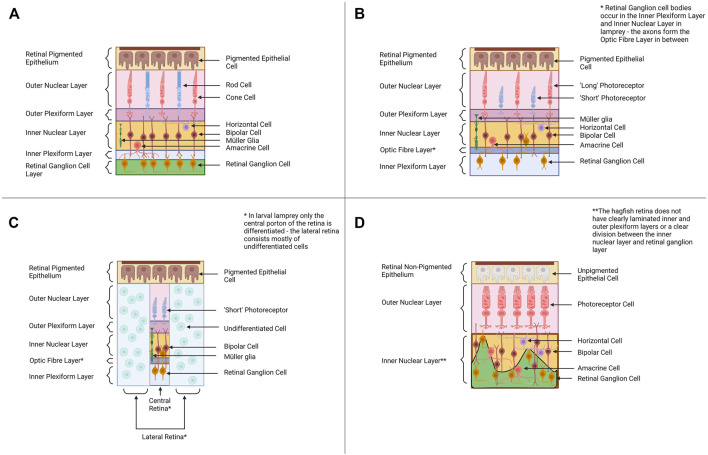
Retinal Organization across Cyclostomes and Gnathostomes provides a backdrop for alternative hypotheses of vertebrate eye evolution. **(A)** Across most all gnathostomes, the retina is composed of four distinct cellular layers—the retinal pigmented epithelium, the outer nuclear layer (ONL) containing photoreceptors, the inner nuclear layer (INL) containing bipolar cells and other interneurons and the retinal ganglion cell layer (RGC) containing retinal ganglion cells. **(B)** The adult lamprey retina has a similar organization to the gnathostome retinal plan. The photoreceptors have a distinct morphology and the retinal ganglion cell bodies occur in the inner nuclear layer and the inner plexiform layer rather than forming their own distinct cellular layer. **(C)** The pre-metamorphic larval lamprey retina is distinct from the adult as it has only a small differentiated central retina (contains one type of photoreceptor, bipolar cells, retinal ganglion cells and Müller glia) and a larger undifferentiated peripheral retina. Retinal differentiation and the formation of horizontal and amacrine cells occurs during metamorphosis. **(D)** The hagfish retina is morphologically distinct from lamprey and gnathostome retinae, and this is intriguing when considering its position in the phylogeny (Figure 1A). The retinal epithelium is unpigmented, and the lamination between the presumptive inner nuclear layer and the retinal ganglion cell layers is poor. Despite the reduced organization, molecular studies support the hagfish retina contains all four cellular layers seen in lamprey and gnathostomes (Dong and Allison, 2021). Graphics created using BioRender.com.

The vertebrate eye is an organ that is highly conserved (in structure, function, physiology and development) throughout the vertebrates from fishes to mammals (Lamb, 2013). The eyes of cyclostomes and gnathostomes share the same basic organization and neural wiring, although the hagfish eye is lacking some elements seen in the eyes of other vertebrates (i.e., the lens). In the typical vertebrate camera-style eye, the cornea and lens focus light onto the retina at the back of the eye where light detection (and the initial processing/transmission of light information) occurs (Lamb et al., 2007; Koenig and Gross, 2020). The retina consists of four distinct cellular layers in typical vertebrates (Figure 2A). The outer nuclear layer (ONL) contains photoreceptors (rod cells for dim-light detection and cone cells for bright light photoreception) (Stenkamp, 2015). The inner nuclear layer (INL) contains the cell bodies of various interneurons (bipolar cells, amacrine cells and horizontal cells) involved in the initial processing of light information and transmitting the information from photoreceptors to the ganglion cell layer. The inner nuclear layer also contains Müller glia (cells that provide a supporting role to retinal neurons and a source of stem cells after retinal damage) (Stenkamp, 2015). The retinal ganglion cells (RGC’s) of the ganglion cell layer (GCL) transmit light signals to the brain (the axons of the RGC’s form the optic nerve). Some RGCs contain melanopsin and are intrinsically photosensitive (ipRGC’s) (Ecker et al., 2010; Schmidt et al., 2011). The fourth cellular layer is the retinal pigmented epithelium (RPE) which is adjacent to (and interdigitated with) the photoreceptors. The cells of the RPE provide a supporting role to the photoreceptors by absorbing stray light, phagocytosing dying photoreceptor segments and aiding in the retinoid cycle (Bharti et al., 2006). The retina also contains two synaptic layers with the outer plexiform layer consisting of the synapses between photoreceptors, bipolar cells, and horizontal cells and the inner plexiform layer forming from synapses between the bipolar cells, amacrine cells and retinal ganglion cells (Centanin and Wittbrodt, 2014; Stenkamp, 2015). This is the almost-universal retinal organization found in jawed vertebrates, but the retinal layers of cyclostomes differ (see below).

The eyes of adult lamprey share many morphological and physiological similarities with gnathostome eyes. In lamprey, the adult retina forms the same four cellular layers seen in the gnathostome retina (Figure 2B). All six retinal neuron types characterized in gnathostomes have also been identified in lamprey retinae, as well as Müller glia (Villar-Cheda et al., 2008; Suzuki and Grillner, 2018; Govardovskii et al., 2020). Adult lamprey have bipolar, amacrine and horizontal cells, based on immunocytochemical data (Villar-Cerviño et al., 2006; Abalo et al., 2008). All lamprey species studied to date also have at least two distinct morphological classes of photoreceptors, “long” and “short” (some species have multiple subtypes of long photoreceptors) (Dickson and Graves, 1979). Morphologically both long and short photoreceptors appear cone-like (based on features used to classify gnathostome rods and cones) (Fain, 2020). However, the short photoreceptors are physiologically similar to gnathostome rod cells and are sensitive to single photons of light (Morshedian and Fain, 2015). The long photoreceptors behave like gnathostome cone cells and function best at higher intensities of light. The lamprey retina contains a layer of retinal ganglion cells, but this cellular layer is organized differently than in jawed vertebrates. In gnathostomes the axons of RGC’s travel next to the inner limiting membrane (adjacent to the GCL) whereas in lamprey the RGC axons travel between the IPL and the INL (Fain, 2020). The cell bodies of the retinal ganglion cells lie either in the inner nuclear layer or the inner plexiform layer (the ganglion cell bodies do not lie in a distinct layer). It is currently unknown why the wiring of RGC’s is different in lamprey and how this could affect visual function. The RPE layer appears to be functional in adult lamprey and contributes to debris removal (via phagocytosis) and retinoid cycling as in gnathostomes (Meyer-Rochow and Stewart, 1996).

One important factor to consider when discussing the lamprey retina is that the lamprey eye undergoes dramatic changes throughout ontogeny. In early lamprey larvae, the eye is buried under a layer of skin and only a small central region of the retina differentiates: the central retina produces one type of photoreceptor, bipolar cells and retinal ganglion cells (Dickson and Collard, 1979; Suzuki and Grillner, 2018) (Figure 2C). As the lamprey matures, more of the retina undergoes differentiation. In late larvae an additional region peripheral to the central retina differentiates (producing retinal ganglion cells) (Cornide-Petronio et al., 2015). Finally, during metamorphosis retinal differentiation is completed across the most peripheral parts of the retina and amacrine cells and horizontal cells appear (Rubinson and Cain, 1989; Meyer-Rochow and Stewart, 1996; Abalo et al., 2008). The RPE does not appear to be functional in the larval lamprey (Meyer-Rochow and Stewart, 1996). Outside of the retina, other features of the eye also change throughout lamprey metamorphosis (i.e., the lens completes development during metamorphosis and is only fully functional in adult lamprey) (Meyer-Rochow and Stewart, 1996).

Compared to gnathostome eyes, the hagfish eye must be considered very rudimentary. Their diminutive eyes are covered by a layer of semi-transparent skin (Genus *Eptatretus*) or a layer of muscle (Genus *Myxine*) (Fernholm and Holmberg, 1975). Most studies have concluded the hagfish eye does not contain a lens, although there is a report of a lens from one species, *Myxine garmani* (Kobayashi, 1964); this report has not subsequently been supported by other studies. The hagfish retina is more disorganized (lamination between the layers is less clear) than in gnathostomes but it does appear to contain all the constituents of cellular layers seen in the retinae of other vertebrates (Dong and Allison, 2021) (Figure 2D). Hagfish of the genus *Eptatretus* have photoreceptors, but they are morphologically difficult to distinguish as rods or cones (Fernholm and Holmberg, 1975). Hagfish species from the genus *Myxine* have photoreceptors that are even more distinct from those seen in gnathostomes (the cells have a whorled morphology) ((Holmberg, 1970; Fernholm and Holmberg, 1975; Lamb, 2013). The photoreceptor cells of *Eptatretus stoutii* express rhodopsin, supporting their similarity to gnathostome photoreceptors (Dong and Allison, 2021). Interestingly, physiological studies have demonstrated that hagfish eyes do respond to light, but if hagfish eyes are removed a response to light signals still occurs (Kobayashi, 1964). This indicates hagfish may also utilize extra-ocular photoreceptors (i.e., photoreceptors in the skin) for light detection (Kobayashi, 1964; Patzner, 1978). *In situ* hybridization data suggests the hagfish retina contains bipolar, horizontal and amacrine cells (Dong and Allison, 2021). *PKC-α* (a marker of rod bipolar cells) and *CALBINDIN* (a marker for horizontal cells) have been identified within the interneuron layer of the hagfish retina. The hagfish retina also has expression of *Pax6* (marker for retinal ganglion cells and amacrine cells) and *melanopsin* (a marker of intrinsically photosensitive ganglion cells) (Dong and Allison, 2021). In contrast, Müller glia have not been identified (as of yet) in the hagfish retina. The RPE layer of hagfish (retinal non-pigmented epithelium or RnPE for hagfish from here on) is distinct from that of other vertebrates as it does not contain any pigment (pigment allows for the detection of light direction and pigment cells are usually associated with photoreceptors throughout Animalia) (Bharti et al., 2006). The RnPE does appear to perhaps participate in other traditional functions of the RPE such as removal of cellular debris (evidence of phagocytosis of an outer segment) and retinoid cycling (the RnPE expresses genes for several enzymes that drive retinoid cycling) (Dong and Allison, 2021). The impact of having a non-pigmented retinal epithelium on hagfish vision needs to be investigated further. Whether the unique characteristics of the hagfish eye represent an ancestral state of vertebrate eyes or is due to loss has been debated, and possible evolutionary scenarios will be outlined in Section 3 (Discussion). Despite its rudimentary characters, the hagfish retina appears to share more similarities with the gnathostome eye than traditionally thought, but more work needs to be done to characterize the cell types within the hagfish retina and to determine if they develop using the same mechanisms as in other vertebrates. Comparing the retinogenesis pathways in hagfish, lamprey and gnathostomes may elucidate how the vertebrate retina evolved.

### 2.1 Cyclostomes Have Elements of the Gnathostome Eye Development and Retinogenesis Pathways, but Gene Expression Data Remains Scarce

To gain a clearer understanding of the origins of the vertebrate retina, we can study the development of the retina in the jawless vertebrates. Elements of the retinogenesis pathway that are missing in the cyclostomes (compared to gnathostomes) may have arisen after the jawless vertebrates diverged from the jawed vertebrates (or were lost from cyclostomes after the lineages split). Alternatively, if elements of the retinogenesis pathways are similar in both cyclostomes and gnathostomes, this would support that the retinal organization seen in gnathostomes arose before the split between the two groups. Despite their critical phylogenetic position, eye/retina development in the cyclostomes has not been heavily studied. There are several papers that have examined eye/retinal development within the lamprey and very few studies on hagfish. Comparatively, there have been numerous studies investigating retinal development within the gnathostomes (summarized below) which have allowed us to begin to appreciate the impressive complexity of vertebrate retinal development. Increasing our understanding of cyclostome retinogenesis would allow us to develop a more comprehensive evolutionary perspective on how this complex neural structure (and the developmental mechanisms that produce it) arose.

In gnathostomes, the retina arises as an extension of the forebrain (diencephalon). During neurulation the anterior portion of the neural tube is specified as the presumptive eye field via the expression of several transcription factors (*Pax6*, *Otx2*, *Six3*, *ET*) (Zuber et al., 2003). Hedgehog signalling from the ventral midline specifies *Pax6* to be expressed laterally, allowing for the development of two separate eyes (Yang, 2004). Once the eye field forms, the tissue that will develop as the eye forms from two outpocketings of the ventral forebrain known as the optic vesicles (Cardozo et al., 2019). As the vesicles grow outwards and contact the overlying epidermis, signals from this tissue and the surrounding mesenchyme induce the vesicles to invaginate and form the optic cup (Steinfeld et al., 2013). The optic cup is a bilayered structure with the inner layer becoming the neural retina and the outer layer becoming the non-neural retinal pigmented epithelium (RPE). The patterning of the optic cup into distinct territories (i.e., eye stalk, RPE, neural retina) is mediated by extrinsic signals such as hedgehog, Wnt and FGF’s (fibroblast growth factor) (Yang, 2004; Steinfeld et al., 2013; Cardozo et al., 2019).

The early neural retina consists of a homogenous population of undifferentiated progenitor cells (Centanin and Wittbrodt, 2014). To form into a mature retina with the seven retinal cell types organized into distinct cellular layers, these cells must proliferate and differentiate in a manner that is tightly regulated spatially and temporally. External signals such as FGFs and Shh (sonic hedgehog) help to control whether cells remain in a multipotent proliferative state or begin to differentiate into a particular fate (Yang, 2004). FGFs (FGF3 and FGF8) from the optic stalk region initiate differentiation of RGC’s in the retina by activating expression of the transcription factor *Atoh7* (Martinez-Morales et al., 2005). Hedgehog signalling promotes the expansion of cellular differentiation from the center to the periphery of the retina (neurogenic wave) (Neumann and Nuesslein-Volhard, 2000). Newly differentiated retinal ganglion cells begin to express hedgehog as well, propagating hedgehog signaling within the developing eye (Wang et al., 2005). Notch signaling is critical for maintaining proliferative progenitor cells during retinogenesis (Ahmad et al., 1995; Jadhav et al., 2006; Riesenberg and Brown, 2016). As retinal cells mature, Notch signalling is downregulated and the effect of intrinsic transcription factor programs (see below) allows the cells to take on new fates (Perron and Harris, 2000). The activity of *Gdf6A* and retinoic acid also contributes to a proper balance of proliferating and committed cells within the early retina, ensuring the developing retina generates the correct number of cells (Valdivia et al., 2016).

Transcription factor networks establish the differentiation pathways that retinal cells will follow to take on their final fates. Retinal ganglion cells are the first cell types to form in the retina by activation of *Atoh7* (Martinez-Morales et al., 2005). *Atoh7* subsequently promotes the expression of additional transcription factors, such as *Pou4f2* and *Isl1*, to specify the retinal ganglion cell fate (Mu et al., 2005). Activation of other cell specification programs is initiated after RGCs begin to form. For most retinal cell types, a major (or several major) transcription factors initiate the pathway for a particular retinal cell fate and subsequently activate downstream transcription factor networks to complete specification/differentiation. For example, the production of photoreceptors is initiated by a network of transcription factors including the homeobox genes *rx* (retinal homeobox gene) and *crx* (cone-rod homeobox gene) and the bHLH genes *NeuroD* and *Maf*/*Nrl* (specifically for rod cells) (Chen et al., 1997; Yan and Wang, 1998; Mears et al., 2001; Nelson et al., 2009; Oel et al., 2020). *Chx10* drives bipolar cell specification (activates *Mash1* and *Math3*) (Hatakeyama et al., 2001). *Ptfla*, *Prox1* and *Foxn4* promote horizontal cell fate, whereas a combination of *Ptfla*, *Foxn4*, *Math3* and *NeuroD* expression produces amacrine cells (Inoue et al., 2002; Fujitani et al., 2006; Ohsawa and Kageyama, 2008). Finally, Müller glia fate is dependent upon the expression of *Rax* (*Rx*), *Hes1*, *Hes5*, *Hesr2* and *Lhx2* (Furukawa et al., 2000; Satow et al., 2001; Melo et al., 2016). These seven cell types initially appear during embryonic retinal development, but in several gnathostome groups (and possibly cyclostomes—see Section 2.3) production of new retinal cells continues throughout ontogeny at the periphery of the retina (the ciliary marginal zone).

In cyclostomes the mechanisms for retinogenesis are still mostly unknown. In lamprey the eye undergoes a dramatic transformation during metamorphosis. Prior to metamorphosis the lamprey retina contains two distinct regions: a smaller central retina (adjacent to the optic nerve) with cells that are differentiated, and an expansive undifferentiated peripheral region (remains proliferative) (Villar-Cheda et al., 2008). During metamorphosis the central differentiated region expands at the expense of the peripheral region and additional retina cell types (i.e., amacrine and horizontal cells) appear (Rubinson and Cain, 1989; Villar-Cerviño et al., 2006; Abalo et al., 2008). Interestingly, in adult lamprey the adult eye does not appear to have a proliferative peripheral zone (Villar-Cheda et al., 2008). Compared to gnathostomes, there have been relatively few studies to investigate gene expression in the developing lamprey eye. For example, the signals that initiate retina formation in larval lamprey and the switch from the larval eye state to the adult eye state are still unknown (do FGFs, Wnt and hedgehog signaling drive retinal proliferation/differentiation in lamprey as in gnathostomes?). Expression of FGF receptors have been identified in the eyes of early lamprey larvae but further work is required to determine what (if any) role these receptors play in eye development (Guérin et al., 2009). There have been a few studies exploring the role of transcription factors in lamprey eye development. Three *Pax6* paralogs have been identified in the lamprey genome and are expressed in the eye (Ravi et al., 2019) (Table 1). Yamamoto et al. (2020) identified *OtxA* (homolog to gnathostome *Otx2*—critical for photoreceptor and bipolar cell development), *OtxB* (homolog to gnathostome *Crx*—specifies photoreceptors) and *Chx10* (expressed in gnathostome bipolar cells) expression in the adult lamprey retina. Compared to mice, the lamprey *Crx* and *Chx10* homologs appear to have similar expression profiles to that of gnathostomes (expressed in the photoreceptors of the ONL and interneurons of INL respectively). However, the majority of *OtxA* expression occurred in lamprey photoreceptors whereas gnathostomes also have strong *Otx2* expression in bipolar neurons. The authors suggest *Otx2* function may have shifted between agnathans and gnathostomes, with *Otx2* gaining a role in bipolar cell specification later in gnathostome eye evolution. Investigation of other retinal development genes could allow us to dissect which elements of the vertebrate retinal system may be ancestral for all vertebrates and which arose later in specific lineages. Examining genomic data available in Ensembl and NCBI (National Center for Biotechnology Information), the lamprey genome possesses homologs for other homeobox genes critical for (gnathostome) eye development such as *Six3/6* and *Rx/Rax* (Table 1). Lamprey also have genes necessary for retinal neurogenesis in other vertebrates such as neurodifferentiation factor (*NeuroD1*), *neurogenin*, *Ascl1* and *Atoh7* (Häming et al., 2011; Lara-Ramirez, 2013 (unpublished); Lara-Ramírez et al., 2015; Higuchi et al., 2019) (Table 2). *Ascl1* expression has not been explored closely in the lamprey retina but has been identified in the lens of larval lamprey (Häming et al., 2011). If and how these genes are expressed in the retina is unknown. More detailed expression studies (including experiments manipulating gene expression) are needed to establish if the above genes coordinate lamprey retinogenesis similarly to gnathostome retinal development. If retinogenesis pathways are conserved between lamprey and gnathostomes, this would support that the basic mechanism for vertebrate retinal development evolved in the ancestor of cyclostomes and gnathostomes.

**TABLE 1:** Retinal homeobox genes across representative vertebrates.

Gene	Hagfish	Lamprey	Zebrafish	Mouse	Function
*Pax 6*	Present Dong and Allison (2021); Feiner et al. (2014)	Present Ravi et al. (2019)	Present Feiner et al. (2014)	Present Feiner et al. (2014)	Acts as a master regulator of eye development, specifies eye field, regulates timing of retinogenesis, regulates retinal cell multipotency and contributes to specification of multiple retinal cell types Chow et al. (1999); Marquardt et al. (2001); Kozmik (2005); Philips et al. (2005); Oron-Karni et al. (2008); Remez et al. (2017)
*Otx1*	Present^a^ (*OtxC*)	Present (*OtxC*) Yamamoto et al. (2020)	Present Lane and Lister (2012)	Present Martinez-Morales et al. (2001)	*Otx1* contributes to the formation of distinct eye regions (i.e., neural retina vs. RPE) and the proper formation of the neural retina Martinez-Morales et al. (2001); Lane and Lister (2012)
*Otx2*	Present^a^ (*OtxA*)	Present (*OtxA*) Yamamoto et al. (2020)	Present Lane and Lister (2012)	Present Martinez-Morales et al. (2001); Nishida et al. (2003)	*Otx2* contributes to specification of the eye field in the neural plate, formation of distinct eye territories, and proper formation of photoreceptors and bipolar cells Martinez-Morales et al. (2001); Nishida et al. (2003); Lane and Lister (2012)
*Otx5/Crx*	Present^a^ (*OtxB*)	Present (*OtxB*) Yamamoto et al. (2020)	Present^b^ Shen and Raymond (2004)	Present (Crx) Furukawa et al. (1997)	Aids in terminal differentiation of photoreceptors Chen et al. (1997); Furukawa et al. (1997); Viczian et al. (2003)
*Six3*	Present^c^ Oisi et al. (2013)	Present^c^	Present Kumar (2009)	Present Kumar (2009)	Promotes eye field formation, promotes neural retina fate over RPE and together with *six6* promotes neural retina progenitor fate Carl et al. (2002); Kumar (2009); Diacou et al. (2018)
*Six6*	Present?^c^	Present?^c^	Present Kumar (2009)	Present Kumar (2009)	Promotes neural retina progenitor fate alongside *six3* Jean et al. (1999); Kumar (2009); Diacou et al. (2018)
*Rx/Rax*	Present Kon and Furukawa (2020)	Present Kon and Furukawa (2020)	Present Mathers et al. (1997)	Present Furukawa et al. (2000)	Necessary for optic vesicle formation; promotes proliferation of retinal progenitor cells, maintains Pax6 expression, helps specify Müller glia Mathers et al. (1997); Furukawa et al. (2000); Nelson et al. (2009)

a Yamamoto et al. (2020) support homology of lamprey *OtxA*, *OtxB*, and *OtxC* to gnathostome *Otx2*, *Otx5*/*Crx*, and *Otx1* respectively. Higuchi et al. (2019) support the homology of Lamprey *OtxA*, *OtxB* and *Otx C* to hagfish, supporting hagfish also have these homologs.

b Mammalian *Crx* is a highly divergent orthologue of *Otx5* whereas the zebrafish *Crx* gene is believed to be from an independent duplication event (zebrafish have both an *Otx5* gene and a *Crx* gene) (Plouhinec et al., 2003; Shen and Raymond, 2004).

c Hagfish (*Eptatretus burgeri*) and lamprey (*Petromyzon marinus*) appear to have *Six3/6* paralogs, but it is difficult to assign the homologs as closer to a *six 3* or *six6* identity. Oisi et al. (2013) identified a *Six3/6* homolog in hagfish. A TBLASTN search against the hagfish and lamprey genomes in Ensembl identified 3 possible *Six3/6* homologs in hagfish and 3 possible homologs in lamprey (when reciprocally blasted each of these sequences blasted most closely to mouse/zebrafish *Six3* or *Six6*).

**TABLE 2:** Retinal bHLH Genes across representative vertebrates.

Gene	Hagfish	Lamprey	Zebrafish	Mouse	Function
*Atoh7*	None found^a^	Present Lara-Ramirez (2013)	Present Miesfeld et al. (2020)	Present Miesfeld et al. (2020)	Retinal ganglion cell specification Brown et al. (2001); Kay et al. (2001); Wang et al. (2001)
*Ascl1*	Present^b^	Present Häming et al. (2011)	Present Jorstad et al. (2017)	Present Jorstad et al. (2017)	Regulation of Notch signalling during retinogenesis, Müller glia reprogramming to multipotency, specification of bipolar cells Hatakeyama et al. (2001); Nelson et al. (2009); Gao et al. (2021)
*NeuroD1*	Present Higuchi et al. (2019)	Present Higuchi et al. (2019)	Present Ochocinska and Hitchcock (2009)	Present Cherry et al. (2011)	Photoreceptor and amacrine cell differentiation Inoue et al. (2002); Ochocinska and Hitchcock (2009)
*NeuroD4*	None found	None found	Present (*Zath3*) Wang et al. (2003)	Present (*Math3*) Cherry et al. (2011)	Bipolar cell and amacrine cell specification Hatakeyama et al. (2001); Inoue et al. (2002)
*NeuroG*	Present Higuchi et al. (2019)	Present^c^ Higuchi et al. (2019)	Present^c^ Korzh et al. (1998); Jeong et al. (2006); Hufnagel et al. (2010)	Present Hufnagel et al. (2010)	Helps to drive the initial wave of retinogenesis in the retina (in mammals) Hufnagel et al. (2010)

a *Atoh1* homolog was the top result for a TBLASTN search of mouse and zebrafish *Atoh7* against the hagfish (*Eptatretus burgeri*) genome in Ensembl. A reciprocal blast of hagfish *Atoh1* matched mouse and zebrafish *Atoh1* better than *Atoh7*.

b Three hagfish *ascl1* homologs were identified via a TBLASTN search using mouse and zebrafish *ascl1* homologs against the hagfish genome. The reciprocal best hits for each of the three hagfish sequences was *ascl1* sequences from mouse and zebrafish.

c *NeuroG* homologs are present in zebrafish but their function in eye/retina development is unclear compared to mammals. Similarly, *NeuroG* was identified in the lamprey genome but does not appear to be expressed in the eye (Lara-Ramírez et al., 2015).

Very little is known about retinal neurogenesis in hagfish, partially due to the difficult of acquiring hagfish embryos. Examining genomic data for *Eptatretus burgeri* on Ensembl, hagfish appear to have homologs for eye specifier genes such as *Pax6*, *Otx2*, *Six3/6* (Table 1) and retinal neurogenesis genes such as *Ascl1*, *NeuroD* and *Neurogenin* (Table 2). They also appear to have homologs of lamprey *OtxB* (*OtxB* is supported as homologous to gnathostome *Crx* by Yamamoto et al. (2020) and *rx*/*rax* (Kon and Furukawa, 2020). This suggests that hagfish may have some of the basic genetic machinery utilized by other vertebrates for eye/photoreceptor development. Without data on where these genes are being expressed and how they interact with other potential components of the eye/retina developmental pathways, it is difficult to firmly conclude whether retinogenesis in hagfish proceeds similarly to retinal development in gnathostomes. These types of studies would ideally be performed on embryonic hagfish [a research group in Japan has acquired embryonic hagfish—Ota and Kuratani, (2008)].

Another way to study hagfish retinal development in the absence of embryos would be to focus on the ciliary margin (a source of post-embryonic retinogenesis in most other vertebrates, especially teleosts and amphibians—see Section 2.3). Several pieces of evidence suggest the hagfish retina contains a proliferative ciliary marginal zone (CMZ). Photoreceptor cells located near the ciliary margin have an immature morphology (missing outer segments) whereas cells located closer to the center of the retina appear fully differentiated (Dong and Allison, 2021). Additionally, *Pax6* (a gene known to be expressed in the CMZ of other vertebrates) is expressed in the peripheral hagfish retina. This data, along with the fact that hagfish eyes grow larger over ontogeny, suggests the hagfish retina may still be proliferative throughout ontogeny despite the reduced/degenerate appearance of the eyes and retina (Dong and Allison, 2021). Further characterization of hagfish retinal development would be very insightful for studying the conservation of the retinogenesis pathway in vertebrates. Elements of the retinogenesis pathway missing in cyclostomes could point to elements that did not arise until later in the vertebrate lineage.

### 2.2 The Relationship of the Cyclostome Eye Condition to Chordate Photoreceptive Organs

To explore the origins of the vertebrate eye and retinal neurogenesis, one must contextualize the data against visual sensory organs of their closest invertebrate relatives: the non-vertebrate chordates. This group is composed of the lancelet or amphioxus (cephalochordates) and the tunicates (urochordates) (Figure 1A). Both groups have photoreceptive cells, but neither possesses the complex, paired eye structures of the vertebrate visual system (Lamb, 2013). Whether the visual/light sensitive cells of the non-vertebrate chordates are related to vertebrate eyes has been debated (see below). In addition to work examining the morphology and function of these organs, several studies have begun to examine the development of amphioxus and tunicate photoreceptive organs, allowing for comparisons to be made to retinal neurogenesis in vertebrates. If amphioxus and tunicate photoreceptors are ancestral/homologous to the vertebrate eye, they could be used to study the early transitional stages leading to the vertebrate eye. The developmental processes driving neurogenesis in non-vertebrate chordates could also provide evolutionary context for retinogenesis in vertebrates.

Several studies have suggested the vertebrate ancestor may have had eye structures/photosensitive organs resembling those seen in the extant non-vertebrate chordates. Examining the closest relatives of the vertebrates the cephalochordates (amphioxus) and urochordates (tunicates), these organisms only possess simple photoreceptive cells/organs (not organized into an eye) used for simple light-guided behavior (i.e., phototaxis). Amphioxus possess four distinct regions that carry photosensitive properties: the frontal eye, lamellar body, Joseph cells and dorsal ocelli (Pergner and Kozmik, 2017). The frontal eye and the lamellar body are of particular interest as they contain ciliary photoreceptors (vertebrate photosensitive organs also contain ciliary photoreceptors). Several studies have proposed homology between the frontal eye of amphioxus and the lateral eyes of vertebrates on the basis that both structures contain similar cell types and gene expression profiles. The frontal eye contains a pigment cell directly adjacent to a row of photoreceptors that express two distinct amphioxus ciliary opsins (*op1* and *op3*) (Vopalensky et al., 2012) (Figure 1B). This organization has been compared to the photoreceptors and adjacent RPE layer of the vertebrate retina. In addition, developing amphioxus pigment cells express *Mitf* and *Tyrosinase* (as does the developing vertebrate RPE) and amphioxus photoreceptors express *Otx2* and potentially amphioxus-*rx* (similar to gene expression profile of vertebrate photoreceptors) (Vopalensky et al., 2012). Although the dorsal eye does not possess a layered retinal structure as seen in vertebrates, there are several adjacent groups of neuronal cells that have been hypothesized to be homologous to other retinal cell types seen in vertebrates. One group of neurons within the frontal eye expresses a combination of *pax4/6* and *rx*, which has led to these cells being compared to vertebrate interneurons (Vopalensky et al., 2012). A separate set of frontal eye neurons have been suggested to be retinal ganglion cells or bipolar cells based on their projection to the amphioxus cerebral vesicle (homologous to vertebrate forebrain/midbrain). Based off developmental data the cell types in the amphioxus frontal eye have been proposed to be homologous to cell types in the vertebrate retina, although the photoreceptors themselves are not as complex nor is the frontal eye organized like the vertebrate retina (Vopalensky et al., 2012; Pergner and Kozmik, 2017; Lacalli, 2018). In addition, amphioxus have homologs of genes involved in the phototransduction cascade and the retinoid cycle in the vertebrate retina (Albalat, 2012; Lamb and Hunt, 2017). Ascidian (tunicate) larvae also possess a photosensitive structure, the ocellus (Figure 1C). This organ consists of a pigment cell associated with multiple photoreceptor cells and lens cells (the tunicate lens cells are not believed to be homologous with the lens of the vertebrate eye) (Eakin and Kuda, 1971; Kusakabe et al., 2001; Esposito et al., 2015). Ascidian photoreceptors are ciliary (as are vertebrate photoreceptors) and express a homolog of opsin *Ci-opsin1* (Eakin and Kuda, 1971; Kusakabe et al., 2001). The tunicate homolog of *Rx* helps to form the ocellus and *Onecut* and *Neurogenin* are also expressed in the ocellus (vertebrate homologs of these genes contribute to retinal development) (D’Aniello et al., 2006; Esposito et al., 2015). These features support tunicate photoreceptors may be homologous to vertebrate lateral eye photoreceptors. Tunicate pigment cells have also been compared to the RPE cells of the vertebrates, and as with amphioxus pigment cells they express homologs of *Mitf* and *tyrosinase* (Sato and Yamamoto, 2001; Yajima et al., 2003). Finally, as with amphioxus, the tunicate genome contains homologs of several genes required for phototransduction and retinoid cycling in vertebrates (Kusakabe and Tsuda, 2007; Esposito et al., 2015). The function of these genes within the amphioxus frontal eye and tunicate ocellus would need to be assessed further to support a conserved function between cephalochordates/urochordates and vertebrates, but at the very least certain genetic elements employed in the vertebrate retina may have existed in the chordate lineage prior to the emergence of the vertebrates.

Overall, characterization of cell types and gene expression profiles within the photosensitive structures of extant chordates suggest the basic cell types that contribute to the vertebrate retina may have already arisen within the last common ancestor of all (vertebrate and non-vertebrate) chordates. The vertebrate eye is presumed to have developed from a simpler structure composed of several photoreceptor cells aggregated together and associated with pigment cells. The arrangement of photoreceptive cells and pigment cells is ancient and occurs in photoreceptive organs throughout Metazoans, including invertebrates and the non-vertebrate chordates (Arendt, 2003; Fain et al., 2010). The vertebrate retina, tunicate ocellus, and the amphioxus frontal eye each consist of pigment cell(s) associated with ciliary photoreceptors (contrasting rhabdomeric photoreceptors that underpin vision in many invertebrates). Therefore, the state of photoreceptive organs in amphioxus and tunicates represents a plausible idea of what the early form of the vertebrate eye may have been. However, the laminated vertebrate retina is highly complex and there must have been many modifications to get from a “chordate-like” proto-vertebrate photoreceptive organ to the vertebrate retina (and eye) plan. The transitional stages that would have led from relatively simple clusters of photoreceptive cells to complex vertebrate camera-style eyes would have involved retinal (or photoreceptor associated) cell types increasing in number and variety and to become arranged into distinct layers (Land and Fernald, 1992; Nilsson, 2009). This process may be related to the emergence of sensory placodes and neural crest in the vertebrate lineage. Sensory placodes are embryonic thickenings of the head ectoderm which contribute to the formation of multiple sensory organs (Schlosser, 2006). Placodes first appeared in vertebrates (i.e., are not found in the non-vertebrate chordates) and are believed to have facilitated an increase in the complexity of vertebrate sensory organs by producing high concentrations of sensory cells clustered into particular areas (Schlosser et al., 2014). This arrangement would set up ideal conditions for the formation of sensory organs in the early vertebrate compared to the scattered sensory cells of other chordates. In vertebrates only the lens placode contributes to eye formation and it does not directly produce sensory cells (i.e., photoreceptors), but instead forms the lens (Schlosser, 2006). However, the lens placode does interact with surrounding tissues to induce eye formation by undergoing morphogenetic movement to aid in forming the optic cup and by releasing signals to promote formation of the neural retina (Cardozo et al., 2019). Perhaps the evolution of the lens placode in the vertebrate ancestor created a region of cells specified for a photoreceptor/interneuron fate via a similar signaling mechanism. Once photoreceptors and visual neurons began to develop in higher concentrations near the lens placode, this may have paved the way for the simpler photoreceptive neurons of chordates to become grouped into an organized retina-like structure (this organization may also have been coordinated by the early vertebrate lens placode). Neural crest cells are another vertebrate innovation. They are a population of migratory cells that contribute to multiple structures (especially within the head) in developing vertebrate embryos and are often associated with formation of sensory organs (Yu, 2010; York et al., 2020). Neural crest is important for proper morphogenesis of the developing vertebrate eye—when neural crest is lost the optic cup does not form properly (Bryan et al., 2020). In early vertebrates, neural crest may have allowed for the shift to a more complex eye structure by providing an additional set of cells to coordinate eye/optic cup formation. Ongoing proliferation late into ontogeny could have also contributed to evolution of the cup-like eye morphology. The photoreceptors themselves would have also needed to gain further morphological complexity and efficiency for light detection/processing (vertebrate ciliary photoreceptors are more complex structurally compared to those of tunicates or amphioxus) (Lamb et al., 2007; Pergner and Kozmik, 2017). Assuming the early vertebrate had a phototransduction cascade and elements of the retinoid cycling pathway similar to extant non-vertebrate chordates, these pathways would also require further changes to reach the state seen in extant vertebrates (Kusakabe and Tsuda, 2007; Albalat, 2012; Lamb and Hunt, 2017; Pergner and Kozmik, 2017). Alongside the increase in complexity of visual cells, the vertebrates also have more complex visual pathways in the central nervous system. This had to be coordinated with retinal evolution to allow for the connection between light detection in the eye and visual information processing in the brain to remain linked. There are many factors that need to be considered when postulating about vertebrate eye evolution, but the current evidence suggests certain chordate photoreceptive organs/cell types may be homologous to the vertebrate eye. Therefore, a comparison of the features (and developmental processes producing those features) between the amphioxus frontal eye, tunicate ocellus and the vertebrate retina may provide a more complete image of the state of the ancestral chordate/vertebrate eye and how the photoreceptive organs of these groups arose from it.

### 2.3 Cyclostomes and Post-Embryonic Retinogenesis

**FIGURE 3:**
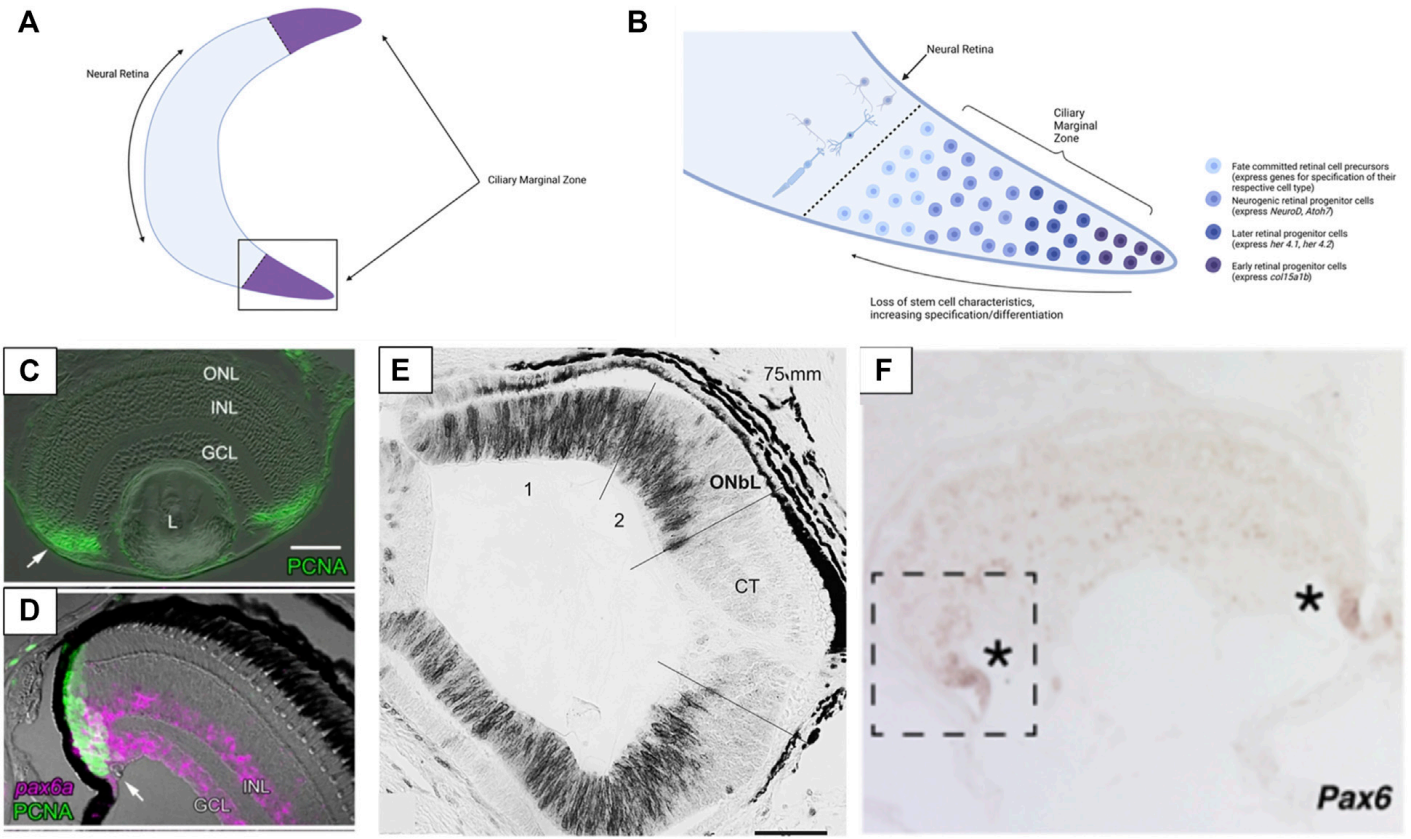
Structure of the ciliary marginal zone (CMZ) in gnathostomes and cyclostomes. **(A)** The ciliary marginal zone is a proliferative region that occurs at the periphery of the neural retina in multiple vertebrate groups (though it is reduced/absent in mammals). **(B)** The boxed section of panel **(A)**. Within the ciliary marginal zone cells closest to the periphery are multipotent stem cells (in groups with a proliferative CMZ) that divide to generate new retina cells. Moving towards the center of the retina the cells of the CMZ begin to express markers of neurogenesis and ultimately become specified and differentiated as one of the main retinal cell types. **(C)** Presence of PCNA (a marker of proliferative cells) in the ciliary marginal zone of zebrafish. **(D)** In zebrafish Pax6 is expressed at the very peripheral margin of the CMZ (Pax6 marks the retinal stem cells and a subset of differentiated INL & GCL cells). **(E)** In lamprey PCNA (black) labelling indicates that cell proliferation also occurs throughout the more peripheral sections of the retina. Once lamprey reach complete metamorphosis the proliferative region of the retina loses PCNA expression (the CMZ-like tissue is not maintained into adulthood). **(F)** The hagfish retina has expression of Pax6 at the most peripheral margin. This is similar to Pax6 expression seen in the CMZ of other vertebrates and supports hagfish have a CMZ (or a CMZ-like tissue) at the margin of the retina. Panels **(C, D)** were adapted from Raymond et al. (2006) (Copyright ^©^ 2006, Raymond et al.; licensee BioMed Central Ltd.). Panel **(E)** was adapted from Villar-Cheda et al. (2008) (Reprinted from Brain Research, Volume 1201, Begoña Villar-Cheda, Xesús Manoel Abalo, Verona Villar-Cerviño, Antón Barreiro-Iglesias, Ramón Anadón, María Celina Rodicio, Late proliferation and photoreceptor differentiation in the transforming lamprey retina, Page 61, 2008, with permission from Elsevier). Panel **(F)** was adapted from Dong and Allison, 2021 (Copyright ^©^ 2021, Dong and Allison, 2021; The Royal Society (UK)). Graphics in Panels **(A,B)** were created using BioRender.com.

Retinogenesis is most thoroughly characterized during embryonic development but in most vertebrates retinogenesis continues robustly in adult animals. The ciliary marginal zone (CMZ) is a region of stem cells that occurs around the periphery of the neural retina in many vertebrate groups (Figure 3A). This proliferative cell population generates new retinal neurons throughout ontogeny (Johns, 1977; Fischer et al., 2014) (Figure 3B). Cells of the CMZ are multipotent and capable of forming any of the retinal cell classes (Raymond et al., 1988). This proliferative zone has been identified in teleosts, amphibians, and birds (the CMZ is relatively limited in mature birds) but is not apparent in mammals (Johns, 1977; Hitchcock et al., 2004; Fischer et al., 2014) (Figure 3C). Despite this it has been noted that some cell populations within the peripheral retina of mammals contain markers related to retinal progenitors and can be induced to take on a progenitor identity in cell culture (Das et al., 2005; Jian et al., 2009). The regressive evolutionary loss of the CMZ in mammals, and perhaps also in sharks (Hernández-Núñez et al., 2021) lends tentative support to the speculation that lampreys have lost the CMZ, as discussed below.

The evolutionary origin of the CMZ is unknown but a putative CMZ has been identified in hagfish (Dong and Allison, 2021). The retinal margin of adult hagfish expresses the CMZ marker *Pax6* and contains immature photoreceptor cells. In addition, hagfish eye size increases with body size (supporting the eye grows throughout ontogeny). These findings contrast earlier assumptions that the hagfish eye is degenerate and degenerating, which would be consistent with a release from selective pressure for processing light/visual information (why maintain an eye with no purpose? Contrast against eye loss in cavefish, as mentioned in Section 1 and Section 3.3). As hagfish possess a proliferative retina past embryonic development, this would suggest the eye maintains an ecological value/significance despite its small size and simple organization. Interestingly, larval lamprey also possess a proliferative retinal region, but the adults do not (Villar-Cheda et al., 2008) (Figures 3D,E). The cells of the peripheral lamprey retina appear to stop proliferating after metamorphosis. This phenomenon has only been documented in one species (*Petromyzon marinus*), so it is unclear if all lamprey undergo loss of retinal proliferation during ontogeny or if it is species-specific. Examining the retinae of other lamprey species and life stages would be necessary to elucidate whether any lamprey have a CMZ and post-embryonic retinal growth or if the absence of adult retinogenesis is consistent across this group. The finding that post-embryonic retinogenesis occurs in hagfish but not lamprey suggests that lamprey either have lost the CMZ during evolution, or hagfish and gnathostomes converged on the CMZ condition. Regardless, the existence of the CMZ (or a CMZ- like tissue) in hagfish is surprising. Moreover, the hagfish eye is an actively growing tissue and together these support that the retina may be utilized by these organisms despite their dim-light habitats.

Research comparing the mechanisms driving adult retinogenesis in cyclostomes and gnathostomes could reveal whether the CMZ of hagfish and gnathostomes arose in a shared ancestor or evolved independently in each lineage. Within gnathostomes, the differentiation of cells from the CMZ appears to recapitulate many of the same mechanisms as embryonic retinal development, but this is an area still under investigation (Link and Darland, 2001; Wehman et al., 2005; Xu et al., 2020). In the most peripheral region of the CMZ cells retain full stem cell characteristics and are multipotent (Raymond et al., 2006). Moving from the periphery towards the center of the retina, CMZ cells become more differentiated (begin to express neurogenic and proneural genes and ultimately become fully specified). Xu et al. (2020) have identified seven groups of CMZ cells that are comparable to groups that arise during embryonic retinal development. The most peripheral group of cells express *fabp11a*, a gene seen in early embryonic retinal stem cells. *Her4.2* expression was found slightly more centrally and this gene expression is similar to that seen in early embryonic retinal stem cells. Even more centrally several genes related to the differentiation of retinal cells (*atoh7*—RGC’s, *vsx*—bipolar cells, *rcn*—photoreceptors, etc.) are expressed. These results suggest that the cells from the CMZ pass through several competence stages across the CMZ before taking on their final differentiated state. A similar sequence of stages was documented from cells developing from the CMZ in *Xenopus* (Perron et al., 1998). It is still unknown how the CMZ functions in jawless vertebrates and whether the retinal margin of hagfish can be considered conserved with the CMZ of gnathostomes. However, if the CMZ of hagfish operates similarly to gnathostomes (i.e., embryonic and adult retinogenesis occur via shared mechanisms) this would support the adult hagfish CMZ as a valuable tissue for studying hagfish retinal development in the absence of embryos.

Müller glia are an additional source of multipotent cells for retinal regeneration in vertebrates late into ontogeny (particularly after injury) (Raymond et al., 2006; Lenkowski and Raymond, 2014). Müller glia are the only non-neural cell population within the retina and serve a supporting role for the various retinal neurons. However, Müller glia can also return to a stem-cell fate to then re-differentiate into neurons after injury (Raymond et al., 1988; Bernardos et al., 2007; Fimbel et al., 2007; Goldman, 2014; Lenkowski and Raymond, 2014). The return to a multipotent state is mediated by several genetic networks including activation of *Ascl1* (Gao et al., 2021). Müller glia have been studied heavily in teleost fish and have also been identified in the lamprey retina (Raymond et al., 2006; Fernández-López et al., 2016). However, there is no evidence for the occurrence of Müller glia in hagfish retinae and this is an area where further investigation is warranted.

## 3 Discussion

### 3.1 Models for Vertebrate Eye Evolution

Given the critical position of hagfish and lamprey in the vertebrate phylogeny, these groups can provide important information for uncovering the history of vertebrate eye evolution. Several scenarios have been proposed to explain how the rudimentary eyes of hagfish fit into the sequence of vertebrate eye evolution and whether the hagfish eye can be considered representative of an “ancestral” state for vertebrates. These models also need to consider that lamprey possess sophisticated eyes (comparable to gnathostomes), yet also form a monophyletic group with hagfish. There are multiple possibilities for how the eyes of lamprey and hagfish diverged (gain or loss of features in specific lineages). Given the existing body of evidence we articulate three alternative scenarios for how the hagfish eye may fit into the narrative of vertebrate eye evolution.

### 3.2 Hagfish Eyes as the Ancestral State

**FIGURE 4:**
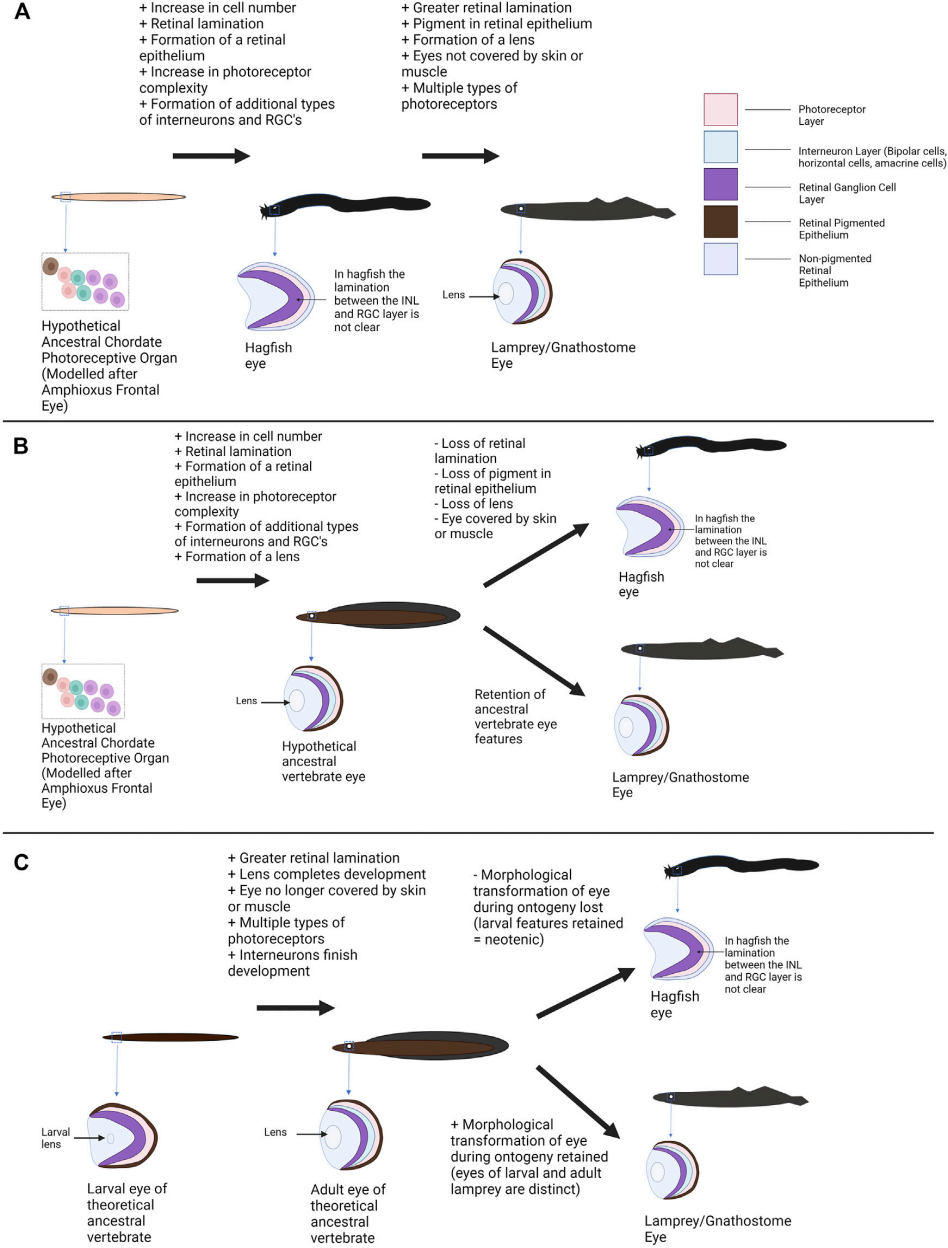
Alternative models of vertebrate eye evolution. **(A)** Hagfish eye as an ancestral state of the vertebrate eye. In this scenario the extant hagfish eye is representative of the ancestral vertebrate eye state (this “primitive” eye still has greater complexity than the photoreceptive structures of non-vertebrate chordates). The lamprey converged upon a more complex eye condition alongside the gnathostomes. **(B)** The hagfish eye as degenerate/regressed. In this scenario the last common ancestor of cyclostomes and gnathostomes has a relatively sophisticated eye. The lamprey and lineage leading to gnathostomes maintained this eye condition whereas the hagfish eye conditions degenerated resulting in extant hagfish possessing rudimentary visual structures. **(C)** The hagfish eye as paedomorphic/neotenic. In this hypothesis, the last common ancestor of gnathostomes and cyclostomes would have undergone a shift in eye morphology during ontogeny with larvae have more rudimentary features and adults having complex eyes. Lamprey (and gnathostomes) maintained this shift to a more complex state. The hagfish no longer completes this transition and the eye seen in adult hagfish represents “juvenile” vertebrate eye features. A mixture of these scenarios may have occurred (see Section 3—Discussion). Graphics created using BioRender.com.

One scenario holds that the hagfish eye represents an ancestral state for the vertebrate eye. This hypothesis interprets the lack of a lens or pigment and the rudimentary features of the hagfish eye/retina as ancestral features related to an early state of eye evolution (Lamb et al., 2007), but no longer has strong support given the monophyly of hagfish and lamprey. Under this model the extant hagfish eye represents a tissue that is akin to a transitional state between the relatively simple photoreceptive cells of other (non-vertebrate) chordates and the more complex eyes of other vertebrate groups (Figure 4A). The hagfish eye (and the hypothetical transitional state) was compared to the pineal organ of other vertebrates (a photoreceptive organ with simpler neuronal organization and photoreceptors than the paired eye) (Lamb et al., 2007; Collin, 2010). This model suggests that features such as the lens of the eye and pigment within the retinal epithelium did not occur in the last common ancestor of hagfish and other vertebrates. The early vertebrate eye would also have few interneurons for processing visual signals compared to later vertebrates (the hagfish condition could be seen as bridging a simpler state comparable to the chordate frontal eye and the complex retina of vertebrates). Again, the ancestral vertebrate retina could be compared to the structure of the pineal gland (photoreceptors directly adjacent to interneurons without additional cellular layers, sufficient for detecting light but not for forming images). Assuming the hagfish eye represents a more “primitive” form of the vertebrate eye, the next stage of eye evolution/organization would then be seen in the lamprey. Although still part of the jawless vertebrate clade, lamprey retinae possess four clearly laminated cellular layers (including the pigmented retinal epithelium) and most of the retinal cell types seen in gnathostomes (including several classes of photoreceptors) (Suzuki and Grillner, 2018). The lamprey eye also contains a lens and pigment in the retinal epithelium. This could be seen as a more derived state of the hagfish eye. In comparison, the hagfish eye may represent a more ancestral photoreceptive organ for circadian entrainment rather than image formation (Lamb et al., 2007).

Assuming the hagfish eye is a baseline/“primitive” state, several ecological drivers could plausibly play a role in the shift towards a more complex eye state in other vertebrates. Changes to the environment and lifestyle of the early vertebrates may have required vertebrates to develop more efficient/effective vision (Collin, 2010; Davies et al., 2012). In addition, if the hagfish eye is compared to the vertebrate pineal this could also represent a shift from use of photoreception in vertebrates for regulating circadian rhythm to image formation and perception of the environment. The addition of a lens would improve visual acuity and photon capture by focusing light onto the retina (Gustafsson et al., 2008). Pigment within the retinal pigmented epithelium would allow for better perception of direction (perhaps related to evolving from a dim benthic habitat into environs with more light) (Bharti et al., 2006). Greater retinal lamination/organization would allow for more efficient processing and integration of visual information (Lamb et al., 2007). Finally, evolution of more complex photoreceptors and multiple opsin classes would allow vision over a breadth of light intensities to evolve (Yokoyama, 2000; Lamb et al., 2007). As deep-sea scavengers, ancient hagfish may have not needed sophisticated vision. However, if other vertebrates took advantage of rich photic niches, good vision would have been critical for avoiding predators and finding prey. This evolutionary pressure would ultimately lead to the formation of a functional and potentially image forming eye.

As hagfish and lamprey constitute a monophyletic clade this scenario is no longer thought to be plausible (Mallatt and Sullivan, 1998; Kuraku et al., 1999; Heimberg et al., 2010; Ota et al., 2011; Miyashita et al., 2019). Lamprey possess relatively sophisticated eyes, and this would imply a large degree of convergence between the lamprey eye state and the gnathostome eye state if the hagfish eye were ancestral. Thus, the last common ancestor of hagfish, lamprey and gnathostomes likely had an elaborate eye with many features familiar from extant vertebrates (Figure 1A). Additionally, although hagfish and lamprey are the only extant Agnathans, several fossils of early jawless vertebrates have been found, and a few studies have investigated the eyes of these specimens. Gabbott et al. (2016) argue that the fossils of both extinct hagfish and lamprey contain pigment. This is a feature that is more consistent with the eye state of extant lamprey than with hagfish and would support an earlier emergence of the “complex” vertebrate eye (i.e., the hagfish eye condition is likely not ancestral for the vertebrate lineage). One limitation of the fossil data is that ocular structures do not generally preserve well. In addition, there can be multiple interpretations for fossil eye data so ideally other lines of evidence (i.e., developmental, genetic, etc.) should be used to support an early origin of these features. The frontal eye of amphioxus and the ocellus of tunicates (argued to be potentially homologous with the vertebrate eye/retina) also contain pigment cells. If the pigment cells of amphioxus and tunicates are ancestral/homologous to the vertebrate RPE (as supported by Sato and Yamamoto, 2001; Yajima et al., 2003; Vopalensky et al., 2012), this would conflict with the hagfish RnPE being the “ancestral condition” for vertebrates (although it does not exclude the possibility that other features of the hagfish eye, such as lacking a lens, may be ancestral). This is an area where an exploration of retinogenesis in cyclostomes may be particularly useful. If it could be demonstrated that the hagfish retina lacks certain retinal developmental pathways seen in other vertebrates, this could support the idea that the hagfish eye is an ancestral condition (rudimentary features due to elements of retinogenesis not being present yet).

### 3.3 Hagfish Eyes as a Product of Regressive Evolution

An alternative scenario/hypothesis is that the hagfish eye represents a degenerate state due to the hagfish adapting to a lowlight environment (the deep-sea). In this model the ancestral hagfish eye would have been similar to that of the extant lampreys or gnathostomes (containing a lens, photoreceptors, pigment). Due to lack of use as they adapted to a dim-light marine scavenger existence, hagfish as a group would have had their eye structures reduced resulting in smaller eyes (covered in skin or muscle), loss of the lens, loss of ocular pigment, and reduced organization/complexity of the retina. As previously mentioned, both living amphioxus and tunicates and at least once species of fossil Agnathan appears to have pigmented cells associated with photoreceptors (Yajima et al., 2003; Vopalensky et al., 2012; Gabbott et al., 2016). This would support that the ancestors of extant hagfish likely had a pigmented retinal epithelium. Fossil agnathans (classified as lamprey) possibly had a lens (Gabbott et al., 2016) and there is an (unconvincing) report of a lens in at least one extant hagfish species (Kobayashi, 1964). These findings suggest that more “complex” features may have existed in ancient cyclostomes. Fernholm and Holmberg (1975) demonstrated that even within living hagfish species the severity of the eye condition can vary with species habitat. Hagfish that live in lower light conditions have more rudimentary eyes (eyes covered by muscle instead of transparent skin, photoreceptor morphology more divergent than in other groups). This phenomenon is not restricted to hagfish as Davies et al. (2009) also showed a light dwelling lamprey species had more complex eyes and a greater variety of photoreceptor subtypes than deeper water species.

There are several genetic and/or developmental mechanisms through which the hagfish eye may have regressed. Other organisms with regressed eyes also tend to live in dim or aphotic environments (such as subterranean and cave-dwelling animals). In these groups (and presumably in hagfish) the eye is no longer useful and therefore the selective pressure to maintain a functional eye is greatly reduced. Genetic drift can then act resulting in the loss of functional genetic machinery to maintain the eye (Jeffery, 2009; Emerling and Springer, 2014). Additionally, the eye/retina is an energetically expensive organ (Yu et al., 2009). Allowing such a structure to become reduced or lost when it is no longer useful allows resources to be utilized on other structures that may be more beneficial for survival. In the deep-sea environment where light is limited and hagfish can rely on olfaction, a fully functional eye may not be worth maintaining (Yu et al., 2009). Finally, if vision is not vital and other structures/systems are expanded, this can result in pleiotropic effects that contribute to eye loss. In cavefish the shift from reliance on vision to other senses (i.e., gustation, mechanoreception), and changes in feeding strategies, has contributed to loss of eyes as the signaling required for proper eye development was altered (Yamamoto et al., 2009; Yoshizawa et al., 2012). Hagfish may have undergone similar evolution: selection for improving other senses (e.g., olfaction) in a dim-light environment could have altered expression of developmental genes with “side-effects” that compromised the developmental pathway for generating an eye. Further work studying the embryonic development of hagfish and potentially manipulating the signalling pathways and genes at play could illuminate whether eye loss is intertwined with the developmental expansion of other sensory systems.

In the wild, hagfish are believed to use vision to a limited extent (if at all). Most hagfish live in deep water, although some species occur in relatively shallow areas where light can reach (Patzner, 1978; Braun, 1996; Martini, 1998). Several studies have suggested that hagfish have limited sensitivity to light. The response of hagfish eyes to light is weak and occurs slowly compared to other vertebrates (Kobayashi, 1964; Patzner, 1978). Interestingly, a behavioural response to light still occurs even if the eyes are removed, suggesting hagfish may have extraocular photoreceptors (Kobayashi, 1964). Light does appear to play a role in circadian rhythm for hagfish and it has been proposed the eye may take on the function of the pineal organ (a pineal has not been identified in hagfish) (Ooka-Souda and Kabasawa, 1995; Lamb et al., 2007; Lamb, 2013). This has led to the standing idea that hagfish are more reliant on other senses (i.e., olfaction) to find food due to their deep-sea environment and living primarily as scavengers. However, some studies suggest hagfish can be predatory (perhaps to a greater extent than previously realized) and hagfish eye morphology does vary with the available light environment (Braun, 1996; Martini, 1998; Zintzen et al., 2011). Fernholm and Holmberg, (1975) noted that hagfish species living in shallower water had more complex eye features than their deeper water relatives. Species in the genus *Myxine* have very reduced eyes covered by muscle whereas *Eptatretus* and *Paramyxine* species have eyes only covered by a translucent layer of skin (some light can reach the eye) (Fernholm and Holmberg, 1975). The photoreceptors of *Myxine* hagfish are more reduced compared to *Eptatretus* and *Paramyxine* as well. This would suggest some elements of eye function remain intact in light living species. Furthermore, it has been reported that hagfish actively avoid (and therefore respond to) light in their environments (Fernholm, 1974). This would support the idea of regression with a transition to dim-light environments. Yet, if regression were the full explanation for why hagfish eyes were reduced it would be odd for hagfish species living in well-lit environments to still have such rudimentary eyes. In *Astyanax mexicanus* (a species with cave-dwelling and surface-dwelling fish) there are populations with both functional vision and varying degrees of eye degeneration (Sifuentes-Romero et al., 2020). However, in hagfish all species surveyed appear to have reduced eyesight. This could be due to the evolutionary timeline of hagfish (i.e., have adapted to this lifestyle over hundreds of millions of years compared to the divergence between surface-dwelling and troglobiont cavefish which diverged several million years ago). Further work is needed to establish to what extent hagfish utilize visual cues in their environment.

### 3.4 Hagfish Eye Regression via Paedomorphosis

An alternate mechanism for regression of the hagfish eye is paedomorphosis. Several groups have compared the hagfish eye to the eye of larval lamprey (Lamb et al., 2007; Collin et al., 2009). Larval lamprey have a relatively simple eyespot, only one type of photoreceptor, and simpler retinal organization/lamination (as described in Section 2) (Suzuki and Grillner, 2018). The larval eyes are also buried under skin and do not emerge until after metamorphosis. These conditions are comparable to the state of hagfish eyes. This has led to the speculation that hagfish eyes may be paedomorphic where their eyes would have ancestrally undergone a transition to a more complex state but heterochronic shifts resulted in the retention of a larval eye state. Extant hagfish have direct development whereas the shift in eye morphology in lamprey occurs during metamorphosis. Regardless, the ancestor of hagfish and lamprey may have had a shift in eye morphology that extant hagfish have lost. The fact that the lamprey retina does undergo some continual development throughout the larval stage (i.e., late-stage larvae have further retinal differentiation than the early larval stage), reduces support for the paedomorphosis model as not all retinal development is restricted to the metamorphic stage (some complexity would still be present in the retina even if the metamorphic stage was lost) (Cornide-Petronio et al., 2015). As developmental data on the retina for embryonic hagfish and lamprey is scarce, it is difficult to support this idea using current evidence. Similarly, it is difficult to use fossil data (fossils of juvenile cyclostomes are quite rare although some have recently been found (Miyashita et al., 2021)). Future work comparing retinogenesis in hagfish and lamprey could greatly help to explore this idea. If hagfish continue to express genes lost early in lamprey retinal development (or do not express genes that are expressed during lamprey metamorphosis) this could support the theory of heterochrony and a paedomorphic hagfish eye. Experiments manipulating expression of the identified genes to see if eye morphology shifts would provide additional support.

## 4 Conclusion

A combination of the above scenarios may have contributed to the evolution of the hagfish eye. The majority of current evidence refutes scenario 1 (the hagfish eye as ancestral). Importantly, the hagfish has been placed into a monophyletic clade with the lamprey (a group with a sophisticated eye/retina plan; Figure 1) (Mallatt and Sullivan, 1998; Kuraku et al., 1999; Heimberg et al., 2010; Ota et al., 2011; Miyashita et al., 2019). Although it is possible the lamprey eye independently converged onto a complex eye condition with the gnathostomes, this scenario is not parsimonious. The hagfish eye also clearly has some features that align with the eyes of lamprey and gnathostomes: despite retinal disorganization the retina still contains the major retinal cell classes, the retinal non-pigmented epithelium still has some function despite having no pigment, and the retina contains a CMZ (Dong and Allison, 2021). These findings support that the hagfish eye has more affinity with other vertebrates than previously thought and suggest the current state of the hagfish eye may be reduced from a more complex state (scenarios 2 and 3). Complexity also arises, because various hagfish eye features may have regressed via separate mechanisms: paedomorphy could possibly account for lack of well-defined retinal lamination in hagfish, but paedomorphy seems untenable for loss of RPE pigmentation because photoreceptors are associated with pigment across all phyla and developmental stages (Bharti et al., 2006; Vopalensky and Kozmik, 2009).

Fossil evidence seems to support the regression theory (fossils appear to have pigment); although a lens was not identified in the fossil hagfish specimen compared to the fossil lamprey (Gabbott et al., 2016). The correlation between the condition of the hagfish eye and the environment (i.e., ambient light levels) also can accommodate the rudimentary eye features being a result of evolutionary regression. The proposal of the hagfish eye as paedomorphic is interesting (especially in the context of the eye phenotype shift during lamprey ontogeny) but requires more work to determine if heterochrony is at play during hagfish development. Determining whether certain eye features are ancestral, or degenerate would be aided by comparison across hagfish species (particularly species that inhabit photic vs. aphotic environments). Future work comparing the retinogenesis pathways of hagfish to lamprey, gnathostomes, and non-vertebrate chordates would also be an enlightening contribution. If complex eye features were indeed lost during evolution there may still be vestiges of associated gene regulatory networks and developmental processes in the hagfish eye (i.e., CMZ in hagfish).

Although the hagfish lineage has been separated from jawed vertebrates for at least 500–600 million years, the hagfish eye has some features that could be considered synapomorphic with gnathostomes and lamprey (i.e., the last common ancestor of gnathostomes and cyclostomes likely had each of the major classes of retinal cell types as these are found in both descendant lineages). Other traits may have been retained from the common ancestor in one lineage and lost in the other or are novel traits that arose in one lineage alone. Work examining gene expression during hagfish eye development and retinal neurogenesis could elucidate which genes were utilized for eye formation in the ancestor of cyclostomes and gnathostomes, versus genes which evolved or were co-opted for different uses in each respective lineage. Overcoming the logistical challenges of studying hagfish retinal development is key. Several research laboratories have bred hagfish embryos which would be an ideal organism for studying hagfish retinal development (Ota and Kuratani, 2008). However, the discovery of a putative ciliary marginal zone is an additional path for exploring retinal growth in adult specimens. In addition to characterizing the retinal neurogenesis pathways, manipulative experiments in hagfish and lamprey to change timing of certain developmental events (testing the heterochrony model) or to recapitulate phenotypes (e.g., altering gene expression in hagfish to recover a more “functional” eye or knocking down genes in lamprey to recreate the hagfish eye condition) are an exciting future avenue for exploring the evolution of retinogenesis in the early vertebrates.

## 5 Outlook and Future Directions

Overall, the early origin of the vertebrate eye remains unclear. The large timespan involved, and understudied nature of the jawless vertebrates (lamprey and especially hagfish) make studying evolution of the vertebrate eye difficult. There is still much to be learned about retinogenesis in agnathans and unravelling the retinal development pathways of these organisms through genetic/molecular/developmental characterization has the potential to clarify the evolutionary origins of this sophisticated sensory organ. The recent increase in availability of hagfish embryos and the discovery of a proliferative CMZ region in adult hagfish creates exciting new opportunities to investigate the emergence of the vertebrate retina and camera-style eye.
